# Idiopathic carpal hyperextension in 12 cats (2018–2025)

**DOI:** 10.1177/1098612X251412747

**Published:** 2026-01-03

**Authors:** Thomas A Marks, Richard L Meeson, Emilie Paran, Graham Hayes, Russell Yeadon, Petra Cerna, Chris Morris, Sorrel J Langley-Hobbs

**Affiliations:** 1 Langford Vets Small Animal Hospital, The University of Bristol, UK; 2 Royal Veterinary College, University of London, Hertfordshire, UK; 3 Kentdale Referrals, Milnthorpe, UK; 4 Lumbry Park Veterinary Specialists, Alton, UK; 5 Colorado State University Veterinary Teaching Hospital, Colorado, USA; 6 Dovecote Veterinary Hospital, Castle Donington, UK

**Keywords:** Idiopathic, carpal hyperextension, pancarpal arthrodesis, carpus, atraumatic, orthopaedic

## Abstract

**Case series summary:**

The aim of the present study was to characterise the clinical presentation, radiographic findings and treatment of idiopathic carpal hyperextension (ICH) in cats. Medical records from six referral centres were retrospectively reviewed to identify cats diagnosed with ICH between 2018 and 2025. Data collected included signalment, clinical presentation, diagnostic findings, treatment approaches and outcome. The study population comprised 12 cats aged 4 months to 12 years 10 months (median 4 years 3 months) with 20 affected carpi. The age distribution showed two peaks: one in younger cats (aged 0–2 years) and another in older cats (aged 9–13 years), with a high proportion of purebreds, notably shorthairs (6/12, 50%). All cats presented with carpal hyperextension without history of significant trauma. Eight cats (66%) were bilaterally affected, one cat initially presented with bilateral disease, while seven developed contralateral involvement 2–36 months after initial presentation. Common clinical findings included a palmigrade stance (12/12, 100%), reduced weightbearing (8/12, 66%) and carpal swelling (2/12, 16%). Increased angulation of the antebrachiocarpal joint was seen on all stressed radiographs, and mild to moderate periarticular soft tissue thickening was seen in most cases (7/11, 63%). All cats were initially managed conservatively (rest and analgesia, mainly meloxicam); four carpi treated conservatively showed an improvement of hyperextension and weightbearing; the outcome was unknown in the remainder of the carpi treated conservatively. In four cats (eight carpi), bilateral pancarpal arthrodesis (PCA) surgery was performed: a single session in one cat and staged sessions in the other three cats. Major complications occurred in two cats, with one requiring revision surgery. All carpi treated with PCA achieved good long-term outcomes.

**Relevance and novel information:**

ICH represents a clinical entity in cats characterised by carpal hyperextension without a history of trauma, frequent bilateral involvement, breed predisposition for shorthair cats and a biphasic age presentation. There is a potential for delayed contralateral limb involvement and a variable response to treatment; therefore, long-term monitoring is recommended.

## Introduction

The feline carpus is a multi-compartmental unit consisting of seven carpal bones, three joint levels and several smaller intercarpal joints.^1^ The joint levels include the antebrachiocarpal (ABC), middle carpal and carpometacarpal (CMC) joints. Individual carpal bones contribute to several intercarpal joints.^2^ The joint capsule, palmar fibrocartilage and palmar ligaments are passive stabilisers of the feline carpus. The palmar and dorsal surface of the joint are stabilised by short intra- and extra-articular ligaments.^3,4^ The carpal flexors, carpal extensors, digital flexors and digital extensors are active stabilisers of the feline carpus.

Carpal hyperextension (CH) is reported to be caused by a focal disturbance of the carpal joint’s stability, usually due to trauma, leading to damage of the palmar ligaments, flexor retinaculum and palmar fibrocartilage, resulting in loss of palmar support, instability and potential luxation or subluxation of the joint.^2–4^ This may lead to joint effusion and osteoarthritis if left untreated.^2^ CH has been reported in many species, including dogs.^5–7^ There are important species differences in anatomy and biomechanics between the dog and cat; in particular, cats have a greater range of antebrachial motion and supination.^4^

CH in cats has been previously reported in association with high-rise syndrome^8–10^ and road traffic accidents.^11–13^ The reported prevalence of carpal injuries in one epidemiological study of feline high-rise syndrome was 29%.^14^ In cats, unlike dogs, CH appears to be less common than other traumatic orthopaedic injuries.^15^ In cats, the ABC and CMC joints are most frequently affected by hyperextension injury;^13,16,17^ the height of the fall may correlate with the joint that is injured.^16^

Although the aetiology of CH in cats is primarily considered to be traumatic, with environmental factors playing a significant role,^2–4^ there is a lack of literature describing idiopathic carpal hyperextension (ICH) syndromes in cats. In contrast, dogs that present with CH with no known trauma are typically cases of metabolic arthropathies, immune-mediated arthropathies or degenerative conditions of the ligaments, particularly in Rough Collies.^18–23^ Atraumatic cases in dogs are typically bilateral.^24^ One relevant study documented feline non-erosive immune-mediated polyarthritis in 20 cats, where five (25%) cats developed ligament laxity, including two cats with bilateral carpal laxity presenting with palmigrade stance.^25^

The relative rarity of CH in cats may explain the lack of data regarding signalment, presenting signs and outcome. Cats with traumatic carpal hyperextension (TCH) will typically present with an acute-onset mild to complete non-weightbearing thoracic limb lameness, a palmigrade stance and a palpable joint effusion.^17^ A palmigrade stance can be categorised by a carpal extension greater than 20°–30°; normal extension is 15° in the cat.^26,27^ Ligamentous instability can be assessed in the sedated patient by applying dorsopalmar stress to the three joint levels.

Various treatment options have been described for managing CH in cats. Conservative management using external coaptation of the carpus has been proposed but has been associated with a high rate of recurrence.^17^ Surgical interventions, including partial carpal arthrodesis, which preserves some joint mobility while stabilising the affected area, have been reported.^28^ Greeff et al^26^ described the use of combined temporary trans-articular internal and external skeletal fixation, without performing an arthrodesis, thereby providing joint stabilisation while maintaining carpal mobility. In more severe cases or when other treatments have failed, pancarpal arthrodesis (PCA) provides complete joint stabilisation at the cost of any joint motion.^29,30^

This paper presents a case series of cats with CH with no known trauma or suspicion of an underlying inflammatory cause. The study objectives were to summarise the current understanding of CH in cats, to explore the possibility of a previously undescribed idiopathic aetiology (ICH), to identify commonalities and differences in a small population of cats with suspected ICH, to provide a descriptive analysis of outcomes in cats with this syndrome, and to describe and compare various management strategies. Our goal was to increase awareness of this condition and inform future diagnostic and treatment approaches.

## Case series description

Clinical record databases at six contributing institutions were reviewed for cases. A total of 12 cases were identified, with one institution contributing five cases, two contributing two cases and three contributing single cases. Inclusion criteria required all cases to be diagnosed with a palmigrade stance compatible with CH. Exclusion criteria included any confirmed history or radiographic evidence of a traumatic aetiology.

Information recorded from the clinical records included signalment, history, clinical signs, radiography reports, results of joint fluid analysis, concurrent orthopaedic disease, treatment and follow-up. All imaging reports were performed by a European College of Veterinary Diagnostic Imaging (ECVDI)/American College of Veterinary Radiology diplomate or resident under supervision of a diplomate. All surgery was performed by a European College of Veterinary Surgery/American College of Veterinary Surgeons diplomate or resident under supervision of a diplomate or a Royal College of Veterinary Surgeons advanced practitioner. Owners of cats were contacted by their respective centres to collect medium- to long-term follow-up of outcomes. Ethical approval was obtained from Bristol University’s Animal Welfare and Ethical Review Body (reference VIN-24-032). All patient details were anonymised.

Data analysis was conducted using Microsoft Excel. The dataset’s main features were analysed using central tendency measures (such as mean, median and mode). Data were compiled in an Excel spreadsheet to enable assessment of the data’s shape, identify outliers and compare different data groups to uncover patterns and trends.

## Results

### Signalment

**Figure 1:**
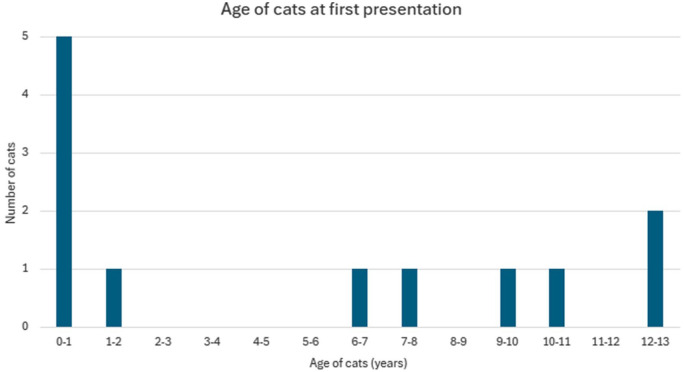
Age distribution of the 12 cats with idiopathic carpal hyperextension at first presentation

A total of 12 cats were classified as having ICH (Table 1). The age at which cats were first noted to be lame ranged from 4 months to 12 years 10 months (median 4 years 3 months). The age distribution showed two distinct peaks: one in younger cats (six cats aged 0–2 years) and another in older cats (four cats aged 9–13 years) (Figure 1). The majority of cats were purebred (n = 9), with the most prevalent breed being shorthair types (British Shorthair, British Blue and American Shorthair, n = 6). Six cats were male and six were female. Nine cats were neutered and three were entire at the time of initial lameness. Seven cats lived exclusively indoors and three predominantly indoors but were allowed outdoors under supervision or on a harness. Two cats were allowed outdoors unsupervised.

**Table 1:** Summary of the signalment, age at onset of presenting signs and limb affected for each cat with idiopathic carpal hyperextension

Cat	Sex	Weight (kg)	BCS/comment on weight	Breed	Indoor/outdoor status	Age at first clinical signs	L/R
1	ME	4.5	Overweight	BSH	Indoor only	10 months	L
–	–	–	–	–	–	3 years 5 months	R
2	FS	5.2	Overweight	DSH	Indoor mostly	7 years	L
–	–	–	–	–	–	7 years 5 months	R
3	FS	4.55	6/9	Persian Cross	Indoors and supervised outdoors	9 years 11 months	R
–	–	–	–	–	–	10 years 1 month	L
4	MC	5.8	6/9	ASH	Indoors only	10 years 1 month	R
–	–	–	–	–	–	10 years 8 months	L
5	FS	3.85	6/9	BSH cross	Indoors only	4 months	L
–	–	–	–	–	–	8 months	R
6	FE	3.8	Unknown	BSH	Indoors/outdoors	6 months	L only
7	FS	3	Unknown	BSH	Indoors/outdoors on harness/supervised	8 months	R only
8	MC	7	Unknown	BSH	Indoors only	10 months	R
–	–	–	–	–	–	Unknown	L
9	ME	4.3	6/9	BSH	Indoors only	1 year 7 months	L only
10	FS	3.8	Unknown	Burmese	Indoors/outdoors	6 years 11 months	R only
11	MC	6.7	7/9	DLH	Indoors only	12 years 2 months	L
–	–	–	–	–	–	12 years 4 months	R
12	MC	9.0	5.5/9	Maine Coon	Indoors only	12 years 10 months	R and L

ASH = American Shorthair; BCS = body condition score; BSH = British Shorthair; DLH = domestic longhair; DSH = domestic shorthair; FE = female entire; FS = female spayed; L = left; ME = male entire; MC = male castrated; R = right

### Presentation

**Figure 2:**
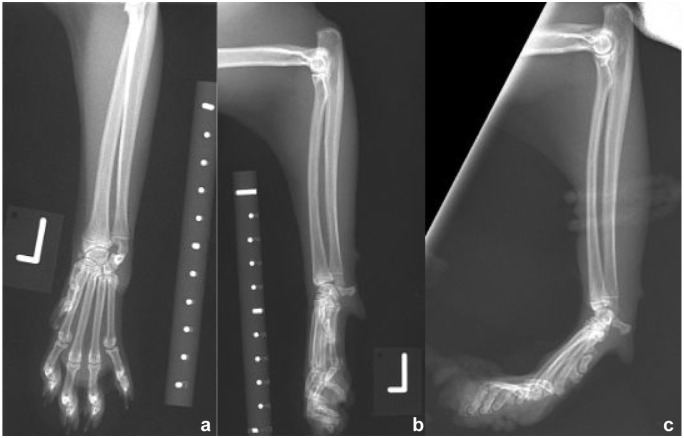
(a) Craniocaudal and (b) mediolateral neutral left carpal radiographs, and (c) mediolateral stressed hyperextended view of the left carpus of cat 1, a male entire British Shorthair cat. There is a left carpal hyperextension with an angle of 50° centred at the antebrachiocarpal joint and mild hyperextension of the metacarpophalangeal and interphalangeal joints. Radiographs taken at 15 months of age

**Figure 3:**
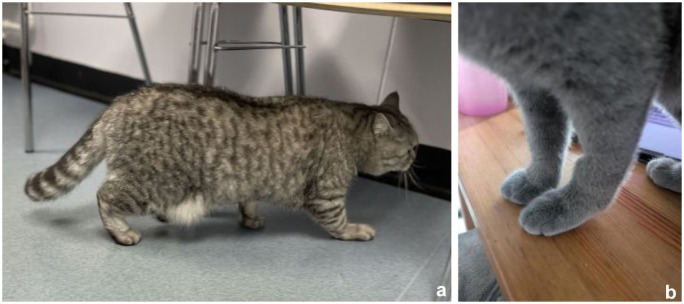
(a) Cat 1, a male castrated British Shorthair cat aged 3 years 5 months with right carpal hyperextension (CH). The left forelimb had been arthrodesed when the cat was 15 months old. (b) Cat 5, a female spayed British Shorthair crossbreed aged 6 months with CH in the left forelimb. This cat subsequently developed right CH (see also Video 1 in the supplementary material)

All 12 cats presented with a palmigrade stance/CH (Figure 2, Table 2). Four cats had CH and normal weightbearing (cats 6, 9, 11, 12). Eight cats (66%) were reported to have reduced weightbearing of the affected limb. The history in 10 cats was of a progressive or insidious onset of forelimb hyperextension. Two cats were reported to be initially holding their limb off the ground when sitting (cats 1 and 4). The hyperextension was reported to be intermittent (not consistently noticed) in two cats (cats 1 and 5) (Figure 3). In total, 11 cats initially presented with unilateral hyperextension and one with acute onset bilateral disease (cat 12). Six cats that presented with unilateral disease later developed bilateral involvement; the time intervals between first and second carpus involvement ranged from simultaneous to a gap of 31 months (mean 8.5) (Table 1) (Figure 3). Of the 12 cats, eight (66%) were ultimately bilaterally affected, with the total number of affected carpi totalling 20.

**Table 2:** History and orthopaedic examination findings of the cats with idiopathic carpal hyperextension (CH)

Cat	History	Orthopaedic physical examination findings	Lameness	Affected limb(s) at first presentation
1	LF insidious onset of carpal palmigrade stance, intermittent tremors, NWB at rest and difficulty retracting claws	L: CH, pain on carpal manipulation, video evidence	Lame	L
	RF developed similar insidious onset of palmigrade stance	R: CH, pain on carpal manipulation	Lame	R
2	Progressive bilateral condition beginning with LF palmigrade stance developing over 8 weeks	L: CH, both tarsi mild plantigrade stance	Lame	L
	Mild RF plantigrade stance, progressed to full palmigrade stance 5 months after LF onset	R: CH	N/A	R
3	RF insidious onset with progressive plantigrade stance and lameness, initial carpal valgus	R: CH	Lame	R
	LF developing similar but milder signs with palmigrade stance 3 weeks later	L: CH	Lame	L
4	RF insidious onset lameness with initial NWB at rest progressing to a palmigrade stance	R: CH	Lame	R
	LF palmigrade stance developed after RF surgical intervention, presenting with lameness and NWB when sitting	L: CH	Lame	L
5	Early onset LF intermittent palmigrade stance beginning at 4 months of age	L: CH, video evidence	Lame	L
	RF intermittent palmigrade stance developed at 8 months of age and became persistent	R: CH, video evidence	Lame	R
6	Acute onset LF palmigrade stance with NWB	L: CH	NWB	L
7	R palmigrade stance noted during routine spay procedure	R: CH, click when flex and extends carpus	NWB	R
8	Mild RF lameness, possible history of a jump and onset of acute lameness	R: CH	Lame	R
	LF palmigrade stance developed after cattery visit	L: CH	Lame	L
9	LF tremors and lameness, progressed to L palmigrade stance	L: CH	NWB	L
10	Progressive onset of moderate RF lameness (max 3/5 grade) developed over 2-week period	R: CH, pain on R carpal manipulation	Lame	R
11	2-month duration of palmigrade stance affecting LF	L: CH, no pain or discomfort	NWB	L
	RF developed similar palmigrade stance 8 weeks after LF	R: CH, no pain or discomfort	NWB	R
12	Acute onset bilateral palmigrade stance affecting forelimbs with abnormal hindlimb posture that was mildly plantigrade	Bilateral CH, moderate tarsal and mild R + L carpal effusions	NWB	R + L simultaneous

L = left; LF = left forelimb; NA = not available; NWB = non-weightbearing; R = right; RF = right forelimb

### Physical examination

Common findings on physical examination included a palmigrade or hyperextended stance (all cats), pain on carpal palpation (cats 1 and 10), tarsal abnormalities (including a mild plantigrade stance, cat 2) and bilateral tarsal effusions (cat 12). Two cats had a concurrent plantigrade stance. Cat 4 had a plantigrade stance of the right hindlimb and a gastrocnemius tendinopathy was identified. Cat 12 had bilateral tarsal swellings and a mild plantigrade stance.

### Radiographic examination

In total, 11 cats underwent orthogonal radiography of both carpi at initial presentation. Six cats had radiographic reports available; for five cats, the radiographs were reported retrospectively by an ECVDI diplomate (EP) (Table 3). One cat also underwent a CT scan of the carpi (cat 1). One cat did not undergo diagnostic imaging (cat 9). Seven cats also underwent stressed hyperextended views of 11 joints (Table 3, Figure 3). The carpal angulation on the stressed hyperextended radiographs was measured in nine cats; in one cat, the radiographs were not available for measurement. If 0° represents a straight carpus and greater than 0° is hyperextended, the range of angles in the nine cats was 30°–65° (mean 38°, 15° of extension is considered normal). One cat had a luxation of the carpus at the ABC joint; it subsequently developed hyperextension in the contralateral joint. Hyperextension of the metacarpophalangeal (MCP) and interphalangeal (IP) joints was subjectively present in five cats (cats 1, 2, 4, 6, 8). Luxation of the ABC joint was documented in 1/12 (8.3%) cases. Specifically, case 2 showed caudal luxation of the left ABC joint. Carpal osteoarthritis (periarticular osteophytosis) was documented in 1/12 cases (case 12).

**Table 3:** Details of the radiological features and a summary of additional problems suffered by cats with idiopathic carpal hyperextension (CH)

Cat	Imaging changes on neutral and stressed radiographs and hyperextension angles	Previous/concurrent problems	Blood test comments and abnormal results
1	Stressed radiography: L CH at 50° Mild periarticular soft tissue swelling Subjective concurrent hyperextension of MCP and IP joints R carpus not evaluated with stressed views	Diarrhoea as a kitten for several months	NA
2	Caudal luxation of the L ABC joint Moderate periarticular soft tissue swelling Stressed radiography: R CH at 30° Subjective concurrent hyperextension of MCP and IP joints	Facial pruritus occurred after PCA surgery Treatment: short-term prednisolone usage	High CK (2700 IU/l)
3	Stressed radiography: bilateral CH documented with L ABC joint at 32° and R CH 30° Mild periarticular soft tissue swelling bilaterally	Ovarian remnant syndrome	Moderate neutropenia, marginal increased urea, mild hypercholesterolaemia, mild hypernatraemia
4	Stressed radiography: bilateral CH with R ABC 45°, L at 32° Mild focal swelling noted over the dorsal aspect of the right carpus Subjective concurrent hyperextension of MCP and IP joints (Figure 2)	Arthritis treated with frunevetmab (Solensia) and occasional gabapentin Previous AKI Plantigrade RH: gastrocnemius tendinopathy	BUN 15.6 mmol/l Creatinine 219 µmol/l
5	Stressed radiographs: bilateral CH centred around the ABC joints (radiographs not available to measure) Moderate bilateral periarticular soft tissue swelling	Severe UTI and anorexia for a few days at 6 weeks of age	Bloods within RI
6	Stressed radiography: L CH at 25° with mild focal swelling over the dorsal ABC Subjective concurrent hyperextension of MCP and IP joints R carpus not assessed with stressed views	NA	NA
7	Radiographic evaluation performed without hyperextended stressed views	NA	NA
8	Stressed radiography: R CH 65° and L 30° Hyperextension of the MCP and IP joints (Figure 4)	Conjunctivitis at 11 months	NA
9	No radiographic evaluation performed	Inspiratory noises caused by elongated soft palate Whole body sway – treated with levetiracetam	Mild lymphopenia 1.23, RBC 10.84
10	Radiographic evaluation performed without hyperextended stressed views, periarticular soft tissue swelling present	Corneal sequestrum	NA
11	Radiographic evaluation performed without hyperextended stressed views	AKI after accidental meloxicam overdose Hyperthyroidism at 15 years	Creatinine and urea within RI after recovery from AKI Very mild leucopoenia and lymphopenia Total protein mildly low and CK mildly elevated T4 normal
12	Radiographic evaluation performed without hyperextended stressed views Bilateral mild carpal osteoarthritis Mild periarticular soft tissue swelling Bilateral tarsal periarticular soft tissue swelling	CKD IRIS stage 2, FOPS, coxofemoral arthritis, pancreatitis, intermittent anisocoria, arrhythmia – ECG trace showed inverted QRS complex with no T wave Partial plantigrade stance Tarsal joint fluid cytology showed no obvious cytological evidence of an infective aetiopathogenesis present or any obvious cytological evidence of a significant neutrophilic inflammatory response or neoplasia	NA

ABC = antebrachiocarpal; AKI = acute kidney injury; BUN = blood urea nitrogen; CK = creatinine kinase; CKD = chronic kidney disease; ECG = electrocardiogram; FOPS = feline orofacial pain syndrome; IP = interphalangeal; IRIS = International Renal Interest Society; L = left; LH = left hindlimb; MCP = metacarpophalangeal; NA = not available; PCA = pancarpal arthrodesis; R = right; RH = right hindlimb; RBC = red blood cell; RI = reference interval; T4 = thyroxine; UTI = urinary tract infection

**Figure 4:**
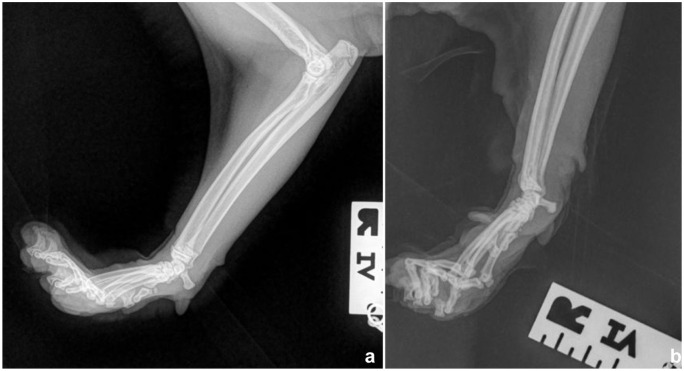
(a) Cat 8: stressed hyperextended radiographs of a 10-month-old male castrated British Shorthair cat showing hyperextension of the left carpus and concurrent hyperextension of the metacarpophalangeal and interphalangeal joints. (b) Cat 3. Stressed hyperextended radiographs of the right carpus of a 10-year-old female spayed Persian-cross cat with carpal hyperextension and normal digital conformation

### Other diagnostic examinations

Soft tissue changes were present in 7/11 (63%) cats. This ranged from mild to moderate periarticular swelling. Soft tissue swelling often involved the entire carpus but was occasionally focal.

Three cats underwent arthrocentesis from affected carpi – one unilateral (cat 1) and two bilateral (cats 3 and 12). In cats 1 and 3, the cell count was considered normal and consisted of predominantly mononuclear cells. In cat 12, the cell count was low and there were 2% neutrophils in the right carpus and fewer than 10% neutrophils in the left. No bacterial organisms were identified in the samples. Synovial fluid was also collected from the tarsus of cat 12; cytological examination was again unremarkable.

Blood analysis was performed in six cats; there were mild abnormalities identified, but no consistent abnormalities to report. Urinalysis was performed in two cats and was unremarkable.

All cats were treated conservatively with analgesia and rest in the first instance (Table 4). Rest protocols varied among cases depending on the severity of CH and owner compliance. Some cats were crate rested while others were restricted to a single room to limit activity levels. All cats were restricted to indoor environments only, with no outdoor access permitted during the treatment period. High-intensity activities, such as playing with toys, jumping and vigorous interaction, were discontinued. Treatment with carpal splinting was attempted in three cats, but this was not well tolerated. No cats were treated with long-term splinting. One cat was treated with a single-angle aluminium carpal brace. Two cats were treated with splinted Robert Jones bandages.

**Table 4:** Summary of conservative and surgical treatment of the cats with idiopathic carpal hyperextension (CH)

Cat	Affected limb(s) at first presentation	Conservative treatment	Conservative outcome	Surgery performed	Surgical outcome
1	L	8 months intermittent meloxicam and rest	Intermittently normal stance but ultimately hyperextension persisted	PCA	22 months: NWB with ongoing CH arthrodesis confirmed on radiographs Occasionally lifts leg Toenails long and needed clipping
	R	NA	NA	PCA	2 months: NWB
2	L	8 weeks meloxicam and rest	Hyperextension persisted	PCA	Mild carpal valgus induced at the time of surgery 5 months: NWB
	R	NA	NA	PCA	3 months: NWB with moderate elbow abduction
3	R	Meloxicam and rest	Hyperextension persisted	PCA single stage	6 weeks: NWB 3 months: improving progressively with minimal lameness on video
	L	3+ weeks meloxicam and rest	Treatment provided significant improvement but intermittent NWB lameness near end of dosing intervals Hyperextension persisted	PCA single stage	
4	R	6 weeks: splint for 10 days – did not stay on frunevetmab (Solensia) Occasional gabapentin	Hyperextension persisted	PCA	3 months: NWB 7 months: R radial fracture at the proximal aspect of the implant Revision surgery with orthogonal plates: good outcome
	L	Frunevetmab (Solensia) Occasional gabapentin	NA	PCA	Metacarpal fracture intraoperative and postoperative swelling 2 months: no lameness
5	L	2 weeks meloxicam, support dressing – did not stay on	Lameness and hyperextension resolved after 7 months	NA	NA
	R	4 months of meloxicam and rest	Hyperextension ongoing	NA	NA
6	L	Splint for 4 weeks	9 weeks after onset of hyperextension ambulating normally with no recurrence of hyperextension	NA	NA
7	R	6 weeks of meloxicam	Lameness resolved at follow-up, ongoing intermittent mild palmigrade stance and NWB at rest	NA	NA
8	R	Rest and NSAIDs	No ongoing lameness at 24-week follow-up, CH resolved	NA	NA
	L	Rest and NSAIDs	NA	NA	NA
9	L	Levetiracetam (for possible neurological issue)	LTFU	NA	NA
10	R	Rest – surgery delayed owing to corneal sequestrum	Regained full mobility at 24-month follow-up, no lameness	NA	NA
11	L	Weight loss	Seen for hyperthyroidism treatment 2 years after CH No mention of carpal issues in clinical notes	NA	NA
	R	Weight loss		NA	NA
12	R and L simultaneous	Daily carpal brace application to the LF for 30 mins to acclimate the cat to the brace and improve ambulation	Mild improvement in the RF at 12 weeks	NA	NA
			Left carpus persistent hyperextension at 12 weeks Ongoing intermittent shifting CH at 9 months R carpal splinting attempted but not tolerated	NA	NA

FU = follow-up; L = left; LF = left forelimb; LTFU = lost to follow-up; NA = not applicable; NSAID = non-steroidal anti-inflammatory drug; NWB = non-weightbearing; PCA = pancarpal arthrodesis; R = right; RF = right forelimb

### Treatment and outcomes

Seven conservatively treated cats had follow-up from 6 weeks to 6 months (Table 4): one cat was lost to follow-up (cat 9); in four cats with four affected carpi (cats 5, 7, 8, 10), the CH was reported to have resolved; and in two cats, the outcome of the second affected leg was unknown or ongoing (cats 5 and 8). In one cat, the lameness resolved but an intermittent palmigrade stance was reported (cat 7).

Four cats (cats 1–4) did not improve with conservative management and were subsequently treated with PCA. Single-stage, bilateral carpal arthrodesis was performed in one cat (cat 3), and unilateral PCA was initially performed in three cats (cats 1, 2, 4), with all three developing contralateral CH 5–31 months later and subsequently requiring contralateral PCA surgery. Eight carpi were arthrodesed in total. In all cases, a 1.5 mm/2 mm hybrid dynamic compression plate/PCA implant was used. Cat 2 experienced a minor complication in the form of a mild carpal valgus; this cat had a chronic carpal luxation at initial presentation. Cat 4 experienced two major complications: a distal radial fracture at the proximal level of the implant 9 months after right PCA and an intraoperative metacarpal fracture 3 months later during PCA of the left joint.

## Discussion

This study describes ICH in cats, a rare condition not previously documented in veterinary literature. Of the cats, 50% were shorthair breeds. The age of the cats ranged from 4 months to over 12 years. All of the cats had increased joint angulation at the ABC joint, and several cats were also observed to have increased extension of the MCP and IP joints. Conservative management was successful in alleviating the CH in four carpi in four cats; another four cats underwent bilateral PCA.

There was a biphasic presentation, with half of the cats aged under 2 years and one-third aged over 10 years. The younger cohort might represent developmental or early-onset forms of the condition. The older cohort could reflect degenerative processes or cumulative microtrauma that eventually leads to clinically significant CH. This age distribution differs from typical TCH, which tends to affect younger cats (aged 2–5 years),^16^ and suggests that ICH may have multiple aetiologies depending on the age of onset.

A notable breed predisposition was observed, with 75% (9/12) of the affected cats being purebred, particularly shorthair breeds, British and American, and one shorthair crossbreed (7/12). This supports a potential genetic component to this condition and contrasts with the lack of breed predisposition reported in TCH.^17^

Significantly, two-thirds of our cases (8/12) presented with or developed bilateral ICH, a feature rarely reported in traumatic disease. The bilateral nature of many ICH cases supports there being an underlying systemic or developmental aetiology. In addition, the delayed development of contralateral ICH in six cats (with intervals in the range of 4–42 months) suggests a progressive condition that may affect both limbs at different times.

Radiography was the primary diagnostic tool in this series, with only one cat undergoing CT. Increased angulation at the ABC joint was noted to be the cause of the hyperextension in all cats where stressed hyperextended radiographs were available. The ABC joint can be affected by TCH, but the CMC and middle carpal joints are also frequently affected by TCH.^16,17^ The joint involvement within the carpus may help to distinguish between ICH and TCH since if hyperextension is present distal to the ABC joint, it is unlikely to be ICH.^17^ Static radiographic views often fail to capture the dynamic nature of carpal instability, particularly when soft tissue structures are primarily affected.^4^

Stressed radiographic views may improve detection of CH but require precise positioning and general anaesthesia/sedation. The inconsistency in availability and technique of stressed radiographs may have impacted how frequently CH was documented radiographically in this series. Furthermore, the complex three-dimensional architecture of the carpus, with multiple joint levels and overlapping bones, can obscure subtle instability on two-dimensional radiographs.^4^ Clinicians should therefore not rule out ICH when radiographs appear unremarkable. The diagnosis should be made on clinical or video examination, and advanced imaging should be considered when available.

The small number of cats that had joint fluid analysis limits the ability to make any conclusions about the significance of the cytological changes. The lack of infectious agents seen on cytology in all samples helps to rule out septic arthritis as a cause; however, culture and PCR techniques have a higher specificity and were not performed in any of the cases. The authors recommend arthrocentesis in all cases to exclude immune-mediated or infectious joint disease.

Differentiating ICH from TCH and other carpal conditions is crucial for appropriate management. Key differentiating factors include the absence of a known traumatic event, the potential for bilateral involvement and the lack of other concurrent trauma-associated injuries. However, it is possible that some cats may experience minor trauma that goes unnoticed by owners, making differentiation difficult. The onset of lameness can also be a differentiating factor, with TCH having an acute onset; many of the cases reported in our series had a more insidious onset and, in some cases, experienced fluctuating or intermittent clinical signs. The bilateral nature of the CH, as seen in the majority of the cats reported here, can also be used to help distinguish ICH from TCH.

Treatment should be individualised, with both conservative and surgical options considered based on severity and the cat’s quality of life. The authors believe that conservative management as the first line of treatment is reasonable in all cases when presented early. This resulted in improvement in four carpi in four cats. The potential for delayed contralateral limb involvement should be communicated to owners.

TCH in cats is purported to be due to ligament rupture.^12,13^ We believe that in some of the cats with ICH the cause may be due to musculotendinous laxity. In the dog, without the support of the superficial and deep digital flexor muscles and tendons, the carpal joint can be hyperextended to approximately 65° (normal angle 0°–10°).^5–7^ Clinical observation of this hyperextension can be seen in cases of digital flexor tendon rupture, with cases of severance or paralysis of the digital flexor muscles and after external coaptation of the carpus, particularly in young dogs and cats.^29^ The presence of concurrent hyperextension of the MCP and IP joints in several of our cats is suggestive of digital flexor tendon laxity. The cats in this series were mainly indoor cats that may tend to exercise less than outdoor cats. British Shorthair cats are a breed known anecdotally for their quiet, placid nature, being relatively inactive and sleeping a lot. Improvement in digital flexor muscle strength may explain the improvement with conservative management in some cases. Persistent hyperextension due to persistent musculotendinous laxity may lead to abnormal forces across the carpus, resulting in pain and inflammation, which contribute further to the lameness. Based on our hypothesis that musculotendinous laxity may contribute to ICH, future studies should investigate whether increased controlled exercise and environmental enrichment to strengthen digital flexor muscles might be beneficial in young cats, rather than the rest and activity restriction that was employed in our cases. Cadaveric biomechanical studies of the feline carpus might provide valuable insights into the pathogenesis of ICH, particularly comparing the effect of digital flexor tendon severance to ligament severance.

The intermittent nature of the hyperextension reported after conservative treatment in three cats would be unusual if the cause was a ligament rupture and may fit more with a diagnosis of musculotendinous laxity. Musculotendinous laxity and CH are seen after external coaptation in young animals and can improve with exercise after cast removal. Metabolic causes of muscle laxity and neuropathy, such as diabetes, should be considered in the diagnosis of CH. Several of the cats had investigations, including blood and urinalysis, to exclude such possibilities, although comprehensive metabolic screening was not performed in all cats.

This study has several significant limitations. The small sample size and multicentre nature of the study restrict the ability to accurately describe the prevalence of the clinical features. The retrospective design introduced inherent limitations, including variability in diagnostic work-up, treatment protocols and follow-up intervals. Advanced imaging was limited, with only one cat undergoing CT and none having an MRI scan, limiting our understanding of the underlying soft tissue and ligamentous abnormalities. Arthrocentesis was not performed as routine, and hence inflammatory arthropathies could not be excluded as a possible aetiological factor. Heterogeneity in treatment approaches makes direct comparison of outcomes impossible, particularly given variable follow-up periods in the range of 8 weeks to 24 months and the cats that were lost to follow-up. Semi-objective outcome measures were not available for the cats for follow-up. Histopathological examination of affected tissues may have helped our understanding of the pathogenesis of ICH but was not possible in any of our cases. In addition, our study population represents a referral caseload, potentially introducing selection bias towards more severe cases. Finally, although no trauma was reported in any case, subclinical or unwitnessed traumatic events cannot be excluded as contributing factors.

## Conclusions

This case series has identified a novel clinical presentation of spontaneous (idiopathic) CH in cats. It is important to identify these cases as being different from traumatic cases, particularly since there is the potential for both resolution and bilateral development. We recommend thorough diagnostic imaging, including stressed radiography with the carpi in hyperextension and the use of advanced modalities, when available, to accurately diagnose and characterise these cases. Investigation into potential systemic, metabolic and developmental causes is recommended. Routine synovial fluid analysis should always be performed. Consideration that the hyperextension may be due to musculotendinous laxity rather than ligament rupture may lead to different treatment regimes and avenues for its prevention. Prospective studies of affected cats with larger sample sizes and longer follow-up periods after both conservative and surgical management would help establish optimal treatment protocols for this poorly understood condition.
